# Characterizing Esophageal Cancerous Cells at Different Stages Using the Dielectrophoretic Impedance Measurement Method in a Microchip

**DOI:** 10.3390/s17051053

**Published:** 2017-05-06

**Authors:** Hsiang-Chen Wang, Ngoc-Viet Nguyen, Rui-Yi Lin, Chun-Ping Jen

**Affiliations:** 1 Graduate Institute of Opto-Mechatronics, National Chung Cheng University, Chia-Yi 621, Taiwan; hcwang@ccu.edu.tw; 2 Department of Mechanical Engineering, National Chung Cheng University, Chia-Yi 621, Taiwan; vietnn.mt@gmail.com (N.-V.N.); jimmyhiei.tw@gmail.com (R.-Y.L.)

**Keywords:** dielectrophoretic impedance measurement (DEPIM), esophageal cancer, cytological stage, admittance

## Abstract

Analysis of cancerous cells allows us to provide useful information for the early diagnosis of cancer and to monitor treatment progress. An approach based on electrical principles has recently become an attractive technique. This study presents a microdevice that utilizes a dielectrophoretic impedance measurement method for the identification of cancerous cells. The proposed biochip consists of circle-on-line microelectrodes that are patterned using a standard microfabrication processes. A sample of various cell concentrations was introduced in an open-top microchamber. The target cells were collectively concentrated between the microelectrodes using dielectrophoresis manipulation, and their electrical impedance properties were also measured. Different stages of human esophageal squamous cell carcinoma lines could be distinguished. This result is consistent with findings using hyperspectral imaging technology. Moreover, it was observed that the distinguishing characteristics change in response to the progression of cancer cell invasiveness by Raman spectroscopy. The device enables highly efficient cell collection and provides rapid, sensitive, and label-free electrical measurements of cancerous cells.

## 1. Introduction

Esophageal cancer is the eighth most common cancer worldwide, and it is the sixth leading cause of cancer death [1]. Esophageal squamous cell carcinoma (ESCC) is one of the most common histological types of cancer lesions in high-risk areas for esophageal cancer, such as Asia, Africa, and South America, and among African Americans in North America [2]. The mortality rates of ESCC remain high because of a lack of reliable morphological and immunohistochemical characteristics in early cancerous stages. Thus, early detection of ESCC is desirable to determine the cancerous stage of the esophageal cancer and to reduce mortality among patients with ESCC [3].

In recent years, many techniques have been applied for the detection, isolation, identification, and characterization of cancerous cells. Label-free and non-invasive approaches have been recognized as valid alternatives to immune-based techniques. Among these approaches, electrical impedance-based techniques have gained a great deal of attention for studying cancer cells because of their attractive advantages with respect to high sensitivity, rapid detection, low cost, and suitability for integrated microsystems [4,5]. Discriminating cells based on impedance spectroscopy provides available information about the physiological properties of cells as a function of frequency [6]. In addition, dielectrophoresis (DEP) is a useful means that can be employed for the rapid manipulation of bioparticles [7]. Microfabrication technology has been adopted to create microelectrode patterns that allow sufficiently large DEP forces to be generated onto cells with low applied voltages. In our previous reports [8,9,10], many rare cancer cells were successfully manipulated by DEP. Combining impedance measurement (IM) with DEP in biosensors is a possible strategy to enhance the sensitivity and reduce the detection period for biological cells [11]. This technique was called the dielectrophoretic impedance measurement method (DEPIM) [3,12]. Faster recruitment of cells by DEP force onto the tips of the microelectrodes results in a higher cell concentration in the gaps between the adjacent electrodes, hence increasing the conductance between the electrodes and reducing the possibility of cell death during measurement.

Several recently published studies have been carried out to identify the different stages of cancerous cells, or the interactions between cancer cells and normal cells [13,14,15]. Most of them require complex surface marker labeling and have high levels of uncertainty, poor efficiencies, high costs and long durations. Among these methods, electrical impedance-based measurement systems have been developed for distinguishing late-stage oral squamous cell carcinomas cell lines from non-cancer oral epithelial cells [14,15]. The cytological stages of cancerous cells can be discriminated by the difference in the kinetics of cell spreading or by the divergence in impedance-based cell index change rate and the cell index between cell types at a given time (at which cells are fully spread) after the cells are cultured on the electrodes. However, these devices still demand a rather long time, and it is postulated that maintaining the optimal conditions for cell growth, including both physicochemical and biological parameters, is required to monitor the evolution of the culture process. In the present study, we describe the design and fabrication of a rapid concentration and highly sensitive measurement biochip using a DEPIM method within interdigitated array microelectrodes. Two ESCC strains, CE81T and CE81T-4, were explored. As a result, these cell lines were differentiated by simply characterizing the admittance of each cell line and by the slope of the curve between the variation of admittance and the cell number. The results demonstrate the potential of the proposed method integrated in the microdevice as a useful analytical approach for cancer research.

## 2. Materials and Design 

### 2.1. Fabrication and Working Principle

**Figure 1:**
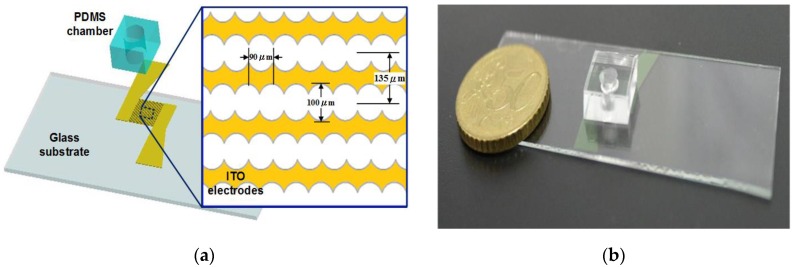
Sketch of the microchip used for cell manipulation and identification. (**a**) The proposed chip design, and illustration of the concentrating and sensing microelectrodes; (**b**) a fabricated chip.

A schematic drawing of the biochip is shown in Figure 1a. The design has three main parts: a glass substrate, interdigitated electrodes and a sample microchamber. In the proposed chip, the structure of circle-on-line microelectrodes [14] is employed, and its dimensions are shown in Figure 1a. The design enables the generation of a positive DEP force on each cell in the chamber when a sinusoidal wave signal with a high frequency is applied to the electrodes. Under the effect of positive DEP forces, these cells will be attracted to the higher gradient regions of the electric field. As a result, the target cells can be captured on the tips of the electrodes. After cell concentrating, the electrical impedance based on the interdigital sensor can be measured within the same device.

Figure 1b shows the image of a fabricated chip using the microfabrication process. A biocompatible material, polydimethylsiloxane (PDMS), was adopted to fabricate the open-top chamber in the microchip for concentrating rare cancerous cells. The microelectrodes were patterned by etching indium tin oxide (ITO) glass substrates using an HCl solution. The PDMS pre-polymer mixture (Sylgard-184 Silicone Elastomer Kit, Dow Corning, Midland, MI, USA) was diluted with hexane, and a PDMS to hexane weight ratio of 1:5 was used. After the PDMS was cured and peeled off, an open-top chamber (4.5 mm diameter) was fabricated using a puncher. The open-top microchamber was subsequently bonded to the ITO glass substrate after 50 s of oxygen plasma treatment in an O_2_ plasma cleaner (Model PDC-32G, Harrick Plasma Corp., Ithaca, NY, USA). The chamber volume was approximately 50 μL.

### 2.2. Theory

Dielectrophoresis (DEP) is known as the force induced on particles under a polarization effect caused by a non-uniform electric field [16]. The DEP force acting on a spherical particle of radius *r_p_* suspended in a medium with permittivity *ε_m_* is given as:

(1) FDEP=2πrp3εmRe(fCM)∇|Erms|2

where *E_rms_* is the root-mean-square of the external electric field, and *f_CM_* is called the Clausius-Mossotti factor. The Clausius-Mossotti factor depends on the electrical properties of the materials, and the frequency of the applied electric field. DEP force depends upon the sign and magnitude of the real part of this factor and is frequently classified into positive DEP (pDEP) if Re(*f_CM_*) > 0 and negative DEP (nDEP) if Re(*f_CM_*) < 0. Therefore, the DEP force varies with the relative polarizabilities of a particle and medium solution, the particles' shapes and sizes, and the frequency of the electric field. As a result, the pDEP cells are guided to the high-electric-field region. In the opposite manner, the nDEP cells are guided to the opposite end. For the construction of the microelectrode used in our research, viable cells were forced by pDEP, because the Clausius-Mossotti factor was defined as approximately 1.0 at a high frequency of 1 MHz [8,17,18].

**Figure 2:**
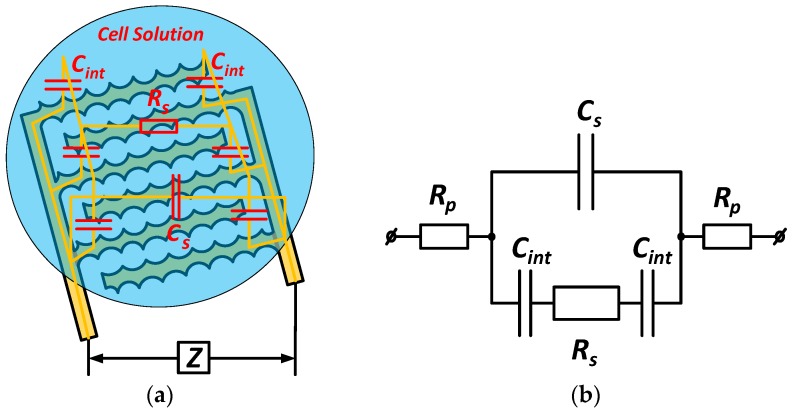
(**a**) Configuration of the interdigitated electrodes structure of the impedance sensor and (**b**) its adapted equivalent circuit model.

To carry out the impedance measurement, a small sinusoidal alternating source was applied for the unknown element at a defined frequency. Then, its electrical impedance could be calculated by Ohm’s law. Resistance and capacitance are the essential components in the impedance measurements of the bioparticles. A typical impedance sensor based on a change of resistance and capacitance leads to changing impedance of the chip. In general, these values depend on the geometric parameters of the electrode structure and the material properties of the interfaces between them. Figure 2a shows the configuration of the impedance sensing structure using the planar interdigitated electrodes. Figure 2b represents the simplified equivalent electrical circuit model of the sensor.

This equivalent circuit model has been used in some studies [19,20,21,22] to determine the total electrical impedance (*Z*) detected in the biochip measurement. The circuit is composed of the solution resistance *R_s_*, the sample capacitance *C_s_*, the electrode-solution interface capacitances *C*_int_, and the parasitic resistances *R_p_*. The parasitic resistance is often the result of the series resistances of the connecting wires. *R_s_* and *C_s_* are related to the conductivity and the permittivity coefficients of the suspended cells and the medium, the geometry parameters of the interdigitated electrodes structure. The impedances at the electrode-solution interfaces are simplified to the double layer capacitances *C*_int_. From Figure 2b, the total observed impedance can be expressed as:

(2) $$Z\left(j\omega \right)=2R_{p}+\frac{1}{j\omega \left(C_{s}+\frac{C_{\mathrm{int}}}{2+j\omega C_{\mathrm{int}}R_{s}}\right)}$$

In a low frequency range, the previous research has shown that the total impedance decreases with the increase of the frequency. The equivalent impedance is determined mainly by the following formulas [21]:

(3) $$Z=\frac{2+j\omega C_{\mathrm{int}}R_{s}}{j\omega C_{\mathrm{int}}}$$

The admittance, which can be derived by the inverse of the impedance, is given as:

(4) $$Y=\frac{1}{Z}=\frac{j\omega C_{\mathrm{int}}}{2+j\omega C_{\mathrm{int}}R_{s}}=\frac{\omega^{2}C_{\mathrm{int}}^{2}R_{s}+j\omega 2C_{\mathrm{int}}}{4+\omega^{2}C_{\mathrm{int}}^{2}R_{s}^{2}}\equiv G+j\omega C$$

in which the conductance (*G*) is expressed as the real part, whereas the reactance (*ωC*) is determined as the imaginary part of the admittance. The variation of the impedance or admittance can be measured to recognize the presence of certain particles in the chip, especially in cancer cells [23]. An accurate electrical impedance-based measurement integrated with other techniques allows us to develop diagnosis devices for certain biological assays of human cells [5]. Thus, this type of measurement is an accordant, label-free and non-invasive approach in the design of cell sensors.

### 2.3. Sample Preparation

ESCC cell lines (CE81T, and CE81T-4), provided by the Division of Gastroenterology, Department of Internal Medicine, Kaohsiung Medical University Hospital, Taiwan, were cultured and used to conduct experiments with the proposed microdevice. The cells were serially passaged as monolayer cultures in Dulbecco’s modified Eagle’s medium (DMEM, Gibco, Grand Island, NY, USA), supplemented with 3.7 g of NaHCO_3_ per liter of medium, 10% fetal bovine serum (Gibco, Grand Island, NY, USA) and 1% penicillin/streptomycin (Gibco, Grand Island, NY, USA). The cell culture dish (Falcon, Franklin Lakes, NJ, USA) was incubated in a humidified atmosphere containing 5% carbon dioxide at 37 °C; the medium was replaced every 1 to 2 days. Cells grown to sub-confluence were washed with phosphate-buffered saline (Biochrome, pH 7.4) and harvested following a 5-min treatment of 0.25% trypsin and 0.02% ethylenediaminetetraacetic acid (Sigma, USA). The cell samples were then suspended in an 8.62 wt% sucrose solution with a measured conductivity of 1.76 × 10^−2^ S/m. For the DEP of cells, the sucrose solution was employed to increase the osmolarity to normal physiological levels. In the Raman spectra of the CE81T and CE81T-4 cell lines, the numerical value behind the “dash” (–) symbol represents the invasiveness of the cancer cells; a higher value indicates greater invasiveness.

### 2.4. Apparatus

**Figure 3:**
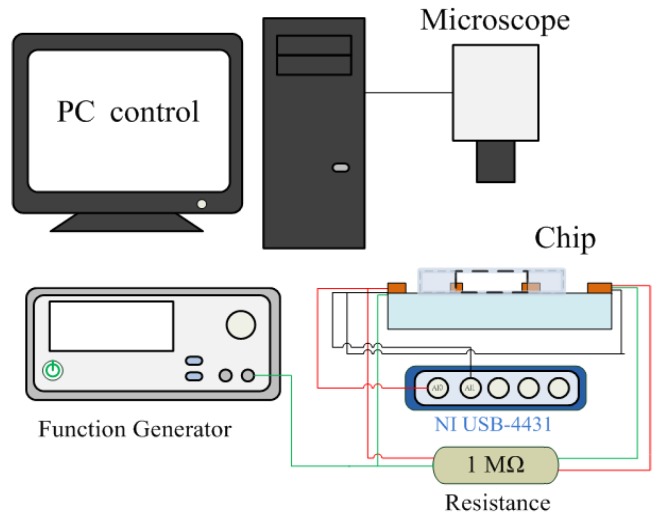
Block diagram of the cell electrical impedance-based measurement and concentration system.

The experimental setup to characterize the device is schematically described in Figure 3. The microelectrodes were driven with a function generator (Agilent 33220A, Agilent Technologies, Inc., Palo Alto, CA, USA) that can provide sinusoidal excitations with a large amplitude and frequency range. DEP manipulation was applied by a peak-to-peak voltage of 5 V at a frequency of 1 MHz, and was created in the cell sample and medium contained in the non-uniform electric field around the electrodes. Therefore, pDEP response would have an effect on each cell suspended in the microchamber. The concentration of the cells was monitored and captured using an inverted fluorescence microscope (CKX41, Olympus, Tokyo, Japan) with a mounted CCD camera (DP71, Olympus, Tokyo, Japan) that was connected to a computer running Olympus DP Controller image software. A specific number of cells was injected in the chamber of a chip for each investigation time. After attracting the cells by the DEP force, the impedance measurement of the sample was then performed and recorded. A voltage signal from a function generator with a frequency of 4 kHz and an amplitude of 1 V_pp_ was used to conduct the impedance parameters measurement. The output signals of the device were collected through a data acquisition card (NI USB-4431). The data were transferred to the PC and were then processed using LabVIEW software to calculate the changes in the impedance of the cell samples.

## 3. Results and Discussion

**Figure 4:**
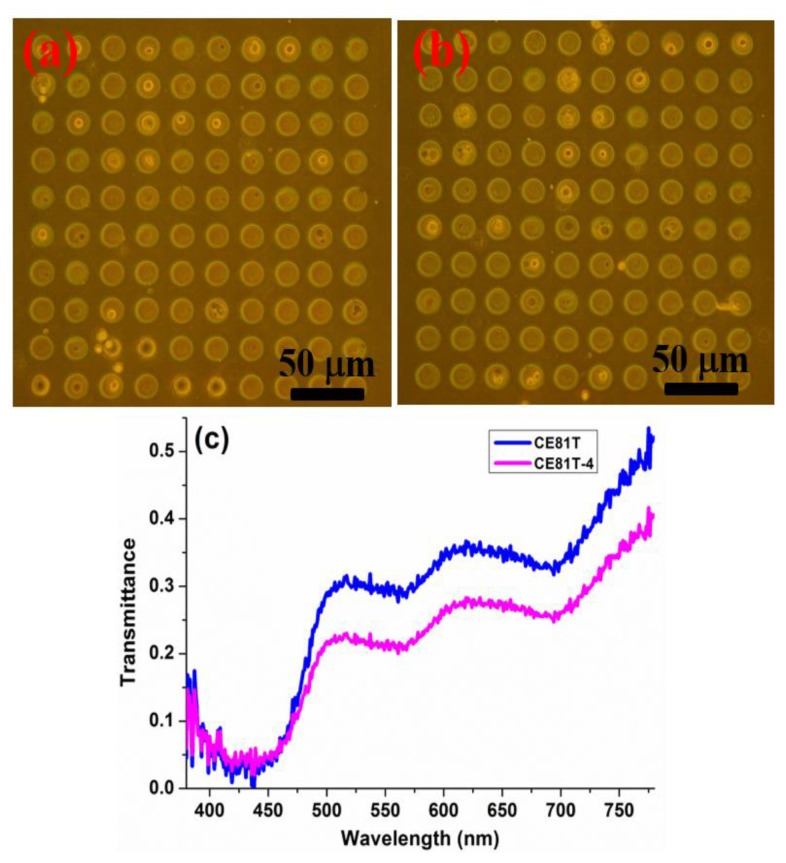
Single-cell array chip images of the two types of esophageal squamous cell carcinoma (ESCC) cells taken by phase-contrast microscopy: (**a**) CE81T and (**b**) CE81T-4. (**c**) Average spectra of CE81T and CE81T-4 from hyper-spectral imaging microscopy (HSIM).

Previously, we reported hyperspectral imaging technology combined with phase contrast microscopy to analyze bladder cancer cells at various stages using a single-cell array chip [24]. The goal of hyperspectral imaging is to obtain the spectrum for each pixel in the image and use such information to find specific objects, identify materials, or conduct optical inspections, and especially medical testing, such as early oral cancer detection [25]. The hyper-spectral imaging microscopic analysis and invasiveness of esophageal cancer cells are detailed in the supplementary materials (Sections S1 and S2). In this work, we adopted our proposed method to analyze ESCC cells at different stages. Detection relies on comprehensive and accurate white-light cystoscopy. In addition to its invasive nature and the potential risks related to the method, white-light cystoscopy has limitations, including difficulties in flat lesion detection, precise tumor delineation to enable complete resection, inflammation and malignancy differentiation, and grade and stage determination. The resolution of these problems depends on the surgeon's ability and experience with the available technology for visualization and resection. From the spectral characteristics of a single cell, we found that the cell spectra at different cancer stages demonstrate changes in the cell's composition. PDMS production and a microfluidics design with biological compatibility were used in addition to a hole array structure to position cells without destroying them for further detection and analysis. The microfluidics single-cell array chip designed in this study is shown in our previous work [24]. Phase-contrast microscopy images of the single-cell array chip after injection with the two ESCC cell lines (CE81T and CE81T-4) are shown in Figure 4a,b, respectively. The cytoplasm is the white round region, and the nucleus at the center of each cell is darkly stained. The differences between the cancer stages of the two types of ESCC cells could not be distinguished under the phase-contrast microscope before this experiment began. The hyperspectral imaging microscopy (HSIM) calculation results for the average spectra of CE81T and CE81T-4 cells without any staining is shown in Figure 4c. Each average spectrum was calculated from the amount of data at 400 points (20 × 20 pixels), taken from 20 cells. The average spectral transmittance increases in the order of CE81T and CE81T-4 because the changes caused by cancer cell progression altered the structure of the cells. More advanced stages of cancer in human ESCC cells have larger nuclear units [24]. The nucleus, which contains DNA and protein, has lower transmittance than the cytoplasm.

**Figure 5:**
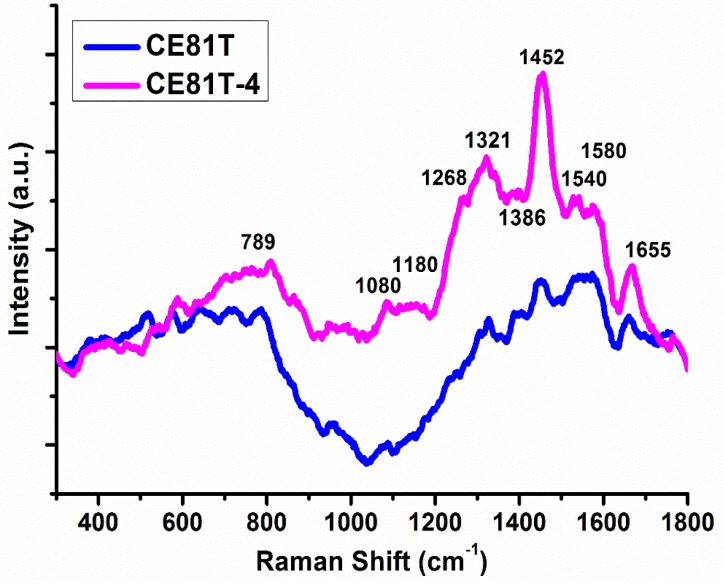
Raman spectra of the two ESCC cell lines (CE81T and CE81T-4).

In addition to detecting ESCC cells using hyperspectral imaging, we also used Raman spectroscopy. Figure 5 shows the Raman spectra of the two ESCC lines (CE81T and CE81T-4). A micro-Raman spectrometer (Renishaw, InVia 1000 system, Renishaw, Gloucestershire, UK) was employed to measure signals at a 633-nm laser wavelength, 8.6 mW average power, 40× magnification, and 4–5 μm spot size. In the measurement process, the sampled cells and buffer were mixed and dropped on the slides, and then 70-nm nanogold particles were mixed with the solution. The nanogold particles may enhance the gain of plasma on the surface, thereby increasing the signal intensity of the Raman spectroscopy [26,27]. The spectra of CE81T and CE81T-4 cells showed considerable Raman peak shifts from 1321 cm^−1^ to 1452, 1540, 1580, and 1655 cm^−1^; these peak shifts are attributed to lipids, amino acids, CH_2_, nuclear acids, tryptophan, and amide C=O, respectively. The corresponding signals became stronger with increasing invasiveness. The above results indicate that CE81T-4 cells were more invasive than CE81T cells.

**Figure 6:**
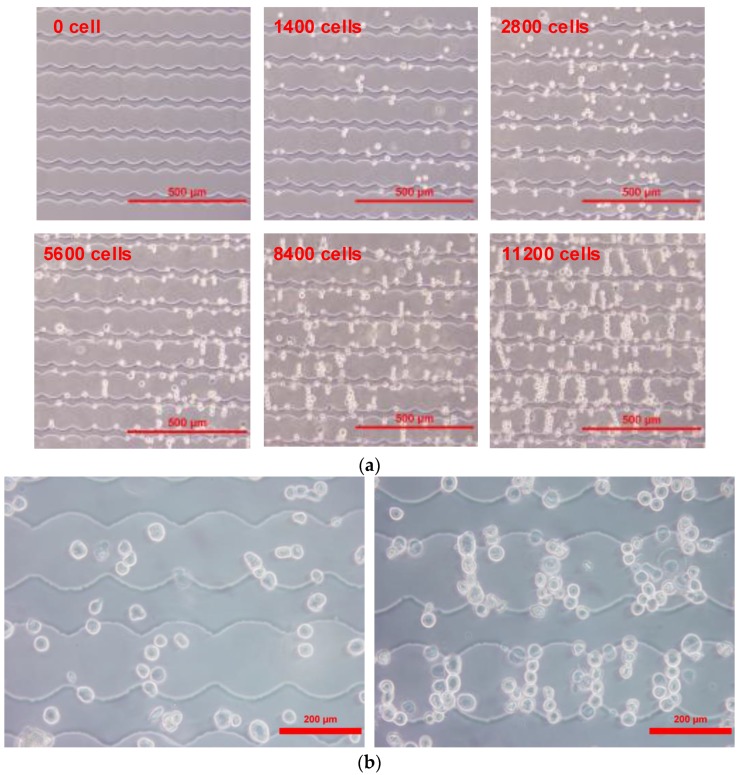
(**a**) Distribution of the CE81T cells after applying positive dielectrophoresis (pDEP) manipulation with various concentrations, and (**b**) the distributions of CE81T-4 cells before and after dielectrophoresis (DEP) manipulation for ten minutes (for 8400 cells).

To evaluate the performance of the microchip herein, samples including the CE81T and CE81T-4 cell lines were examined with different cell numbers ranging from 1400 to 11200 cells. The frequency applied to the microelectrodes was set to 1 MHz in order to generate pDEP effect in our studies. The impedance-based measurement was then performed at a reliable frequency of 4 kHz [28,29]. Each time, DEP manipulation using the sinusoidal signal was applied for 10 minutes, enabling sufficient time to concentrate the cells. This optimal time interval was determined through repeating many previous experiments. The cancerous cells suspended in the chamber were driven by pDEP and attracted to the tips of the microelectrodes. As observed in Figure 6, the microscopic images of cancerous cells were captured with various numbers of cells after the DEP response was completed. Thus, DEP manipulation leads to an increase in the sensing sensitivity of the biochip, due to the significant enhancement of the electrode-solution interface impedance [30]. Next, the conductive and capacitive components of the chip were computed by the real and the imaginary parts of the measured admittance. When applying a measurement signal in the low frequency range, the admittance of the chip was inferred from Equation (4). However, the capacitive component was ignored because the imaginary of its admittance was much smaller than the real value of one at a given cell number. Thus, the magnitude value of the admittance was approximately equal to the conductance. The impedance can be easily calculated by the inverse of the admittance. These tests were repeated many times within a chip.

**Figure 7:**
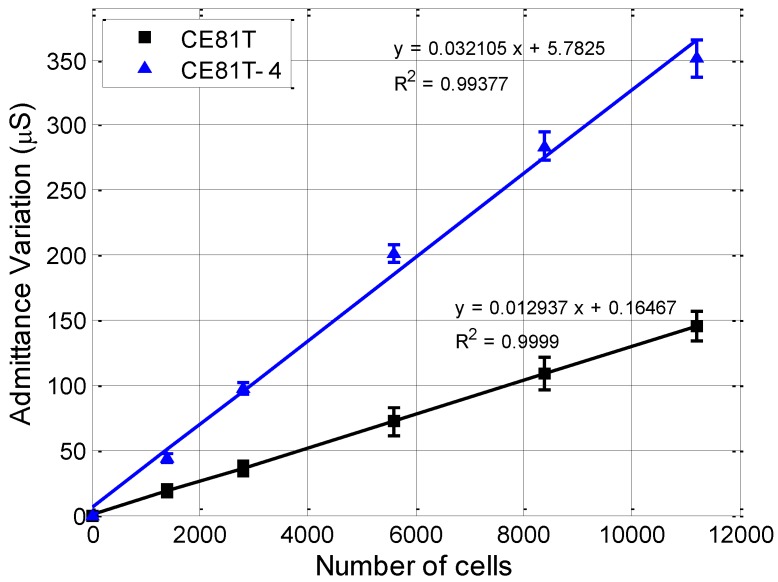
Linear relationships between the change of the calculated admittance and the number of cells for the two types of human ESCC cells. The results show that use of the biochip makes it possible to distinguish different cytological stages of cancer cells by the slope of the graphs.

For each cell type, it is assumed that the initial microchip is the reference sample, in which no cell is injected in the chamber, so that only the medium impedance is sensed. The admittance magnitude of the reference sample was measured to be about 50 ± 5 μS. Next, the impedance of the chip was measured corresponding to the defined number of suspended cells at each investigation time. The admittance change was calculated by the change of the admittance of each test sample in comparison with the reference sample. Figure 7 presents the diagram of the admittance variation of the chip with an increasing number of cells. According to the experimental results, the admittance increased as the number of cells increased. Moreover, the admittance change increased linearly with cell number for all cell types; the slopes are 0.0129 and 0.0321 for the CE81T and CE81T-4 cells, respectively. Regression analysis of the number of observed tumor cells versus the recorded admittance change showed that the respective curves were up to 0.99 for both cell lines. The high linear approximation of the characteristic lines of the admittance variation demonstrates the potential to discriminate the ESCC cells at different stages based on the impedance measurement herein.

In this study, the fabricated chip also indicated the capability of differentiating cancerous cell stages using an electrical impedance-based measurement, which requires a shorter time while the sensitivity still reached the desired effect. As we can see, significant differences in admittance variations could be observed between the two esophageal cancer cell lines, and these divergences between them increase with increasing cell number. At the same cell concentration, CE81T-4 cells generate a higher admittance than CE81T cells. From Figure 6, we clearly observe that CE81T-4 cells tend to cluster more strongly than CE81T cells. Cancerous cells with higher stages seem to interact more closely to each other than the lower stages of the cell line. It appears that the admittance of cancerous cells that are more invasive is larger than the admittance of cancerous cells that are less invasive and normal benign cells. This difference could be the result of cellular morphological variations resulting from changes in the cytoskeleton structure, the cytoplasm conductivity, and, specifically, the membrane topography of cells, because the membrane of cancer cells is related to increased invasiveness and metastatic potential [31]. The increase in cell size may result in a decrease in cytoplasmic conductivity because the intracellular ions are diluted [32]. Differences in the concentration of ion species in the cytoplasm, the membrane permeability and the intracellular volume fraction may lead to differences in cytoplasmic conductivity. In addition, the expression levels of ion channels are different between cell lines, thus promoting either the exit or entry of ions into the cytoplasm. As a result, we can possibly distinguish the different stages of esophageal cancerous cells directly through admittance changes in electrical measurements without calculating cell indices, as well as monitoring cell growth in real-time. Finally, the design also indicates that the DEPIM method could provide a potential approach to distinguish the different stages of cancerous cells.

## 4. Conclusions

A device with microelectrodes that combine both effective concentration and identification of cancerous cells was developed and fabricated in the present work, based on the DEPIM method. The pDEP cells can be focused onto the tips of the microelectrodes because of the generation of high-electric-field regions. Human esophageal cancer cell lines were successfully concentrated by DEP force using an applied peak-to-peak voltage of 5 V_pp_ at a frequency of 1 MHz. Subsequently, electrical impedance-based measurements were performed for various cell numbers of CE81T and CE81T-4 cells with a sinusoidal function signal at an amplitude of 1 V_pp_ and a frequency of 4 kHz. Using the admittance characteristic to recognize and distinguish ESCC cell lines at different cytological stages in a label-free manner was also demonstrated. The microchip has potential as a rapid, label-free, and highly sensitive device that is easy to operate with a low sample volume platform for the detection of cancerous cells. The proposed device can be applied in the fields of clinical diagnostics, biological assays, and biomedicine.

## Supplementary Materials

The following are available online at www.mdpi.com/1424-8220/17/5/1053/s1.
