# Supplementary material for: Characterizing Esophageal Cancerous Cells at Different Stages Using the Dielectrophoretic Impedance Measurement Method in a Microchip
Source: Sensors (Basel). 2017 May 6;17(5):1053. doi: 10.3390/s17051053 (PMC5469658; doi:10.3390/s17051053)
Supplement: Supplementary file 1 [file sensors-17-01053-s001.pdf]

Characterizing Esophageal Cancerous Cells at Different Stages Using the Dielectrophoretic Impedance Measurement Method in a Microchip

Hsiang-Chen Wang[1], Ngoc-Viet Nguyen[2], Rui-Yi Lin[2] and Chun-Ping Jen[2*]

[1]Graduate Institute of Opto-Mechatronics, National Chung Cheng University, Chia-Yi, Taiwan, R.O.C.

[2]Department of Mechanical Engineering, National Chung Cheng University, Chia-Yi, Taiwan, R.O.C.

Corresponding author: Chun-Ping Jen; 168 University Rd., Min-Hsiung, Chia-yi, Taiwan; Tel: +886-5-2729382; Fax: +8865-2720589; Email: imecpj@ccu.edu.tw

S1. Diagnosis of human ECCs at different stages using hyper-spectral imaging microscopy (HSIM)

A biocompatible material, PDMS, was adopted for single-cell-based arrays in the microfluidic chip, as illustrated in Figure S1(a). The main channel, formed on the top PDMS layer, is 15 mm wide, 160 μm in height and 65 mm long. The main channel is divided into eight microchannels, each 1 mm wide and 45 mm long, at the center region. Each microchannel contains fifteen 10 × 10 microwells, 20 μm or 30 μm in diameter and 20 μm deep, on the bottom PDMS layer. The mold masters were fabricated by spinning SU-8 (SU-8 50, MicroChem Corp., Newton, MA, USA) on a silicon wafer to define the microwells and microchannel, respectively. The mold master of the microfluidic channels (around 160 μm in height) was fabricated by spinning SU-8 at 500 rpm for 20 s and then at 800 rpm for 35 s on the silicon wafer. The resist was soft baked on a hotplate at 65 °C for 10 min and then at 95 °C for 30 min. The resist was then allowed to cool to room temperature. The SU-8 was exposed to ultraviolet (UV) radiation at a dose of 200 mJ/cm^2. The post-exposure baking was done at 65 °C for 3 min and then at 95 °C for 10 min. The exposed samples were developed with SU-8 developer for 5 min. The mold master of the microwells (around 20 μm in height) was fabricated by spinning SU-8 at 500 rpm for 20 s and then at 4,500 rpm for 35 s on a silicon wafer. The resist was developed with SU-8 developer for about 2 min after baking and exposure to UV radiation under the conditions

mentioned above. PDMS prepolymer mixture (Sylgard-184 Silicone Elastomer Kit, Dow Corning, Midland, MI, USA) was poured and cured on the mold masters to replicate the patterned structures. Scanning electron microscopy (SEM) images of the SU-8 mold with microwells on the silicon wafer and PDMS replica are shown in Figure S1(b). After peeling off the PDMS replica with the microchannel, the inlet and outlet ports were made by a puncher. The two PDMS replicas were bonded after treatment with oxygen plasma in an O_2 plasma cleaner (model PDC-32G, Harrick Plasma Corp., Ithaca, NY, USA). A photograph of the completed microfluidic chip with tubing is shown in Figure S1(c).

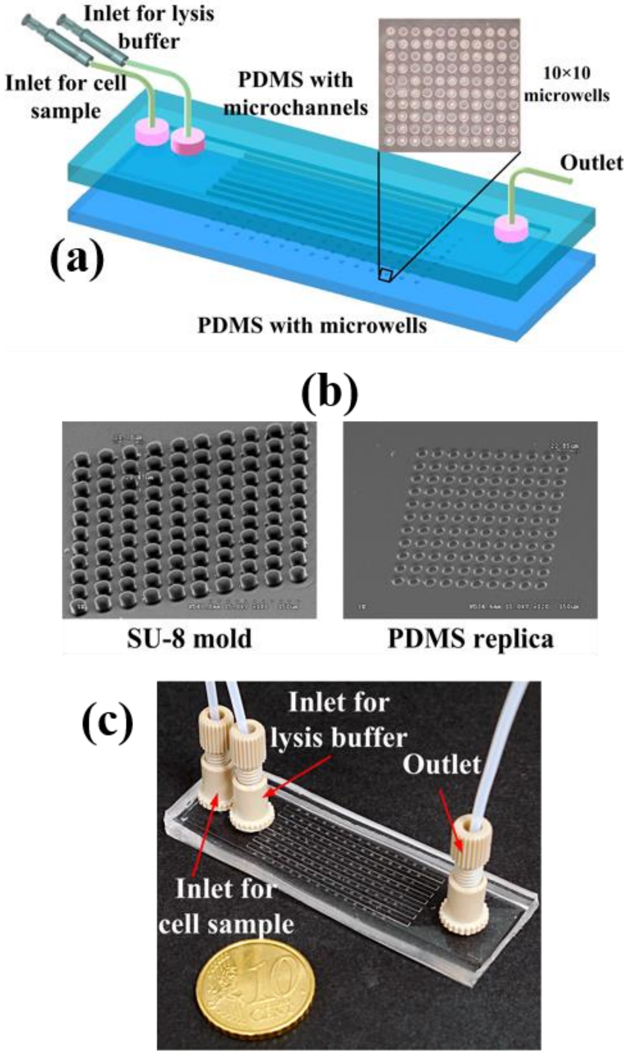

Figure S1 (a) Schematic diagram of the proposed microfluidic chip for single-cell-based microarrays. (b) SEM micrographs of the SU-8 mold on the silicon wafer and PDMS replica. (c) Photograph of the completed microfluidic chip with tubing.

The estimation processes of the average spectra of CE81T, and CE81T-4 cells with HSIM data are illustrated in Fig. S2. HSIM technology was used to obtain the spectrum of each image element of the single-cell array. First, the spectra of the 24 Macbeth color checkers are measured by a spectrophotometer (Konica Minolta CS1000A) under the illumination of a particular uniform artificial light, and the reflection spectrum of each color checker in the visible light region (380nm to 780nm) is obtained. These spectra are arrayed as a matrix, $[D]_{401x24}$, the rows of which are the intensities of the wavelengths at 1 nm intervals, and the

columns of which are the numbers of the color checkers. By determining the eigen-system and applying the principal component analysis (PCA), six eigen vectors that make the greatest contribution are selected to be the basis of the spectral estimation, and arrayed as a matrix $[E]_{6x401}$. The corresponding eigen values of these six eigen vectors $[\alpha]_{6x24}$ can be determined as follows:

$$[\alpha]^T = [D]^T pinv[E] \tag{1}$$

where "pinv" denotes the pseudoinverse of the matrix. Simultaneously, the color checkers are captured by a digital camera under the same illumination condition, for which the output format is sRGB (JPEG image files). The red, green and blue values (from 0 to 255) of each color checker's image are obtained using computer programs, and are then plotted on a scale of R_{srgb}, G_{srgb} and B_{srgb} (form 0 to 1). These RGB values can be transferred into CIE XYZ tristimulus values by the following formula:

$$\begin{bmatrix} X \\ Y \\ Z \end{bmatrix} = [T] \begin{bmatrix} f(R_{srgb}) \\ f(G_{srgb}) \\ f(B_{srgb}) \end{bmatrix} \tag{2}$$

where

$$[T] = \begin{bmatrix} 0.4124 & 0.3576 & 0.1805 \\ 0.2126 & 0.7152 & 0.0722 \\ 0.0193 & 0.1192 & 0.9505 \end{bmatrix} \tag{3}$$

$$f(n) = \begin{cases} \left(\dfrac{\mathrm{n}+0.055}{1.055}\right)^{2.4} & , \mathrm{n} > 0.04045 \\ \left(\dfrac{n}{12.92}\right) & , otherwise \end{cases} \tag{4}$$

Due to the reference white of the sRGB color space being illuminated by a CIE standard light source D65, which is different from the artificial light used for measuring the spectra of the color checkers, these RGB values are corrected for chromatic adaptation by applying CMCCAT2000. Taking the accuracy of spectral estimation into account, color correction of the camera is also required. The reflection spectra measured by the spectrophotometer are transferred into CIE XYZ tristimulus values by using Eqs. 5 to 8. In these calculations, $S(\lambda)$ is the relative spectral power distribution of the artificial light, $R(\lambda)$ is the spectral reflectance of the respective color checker, $\bar{x}(\lambda)$, $\bar{y}(\lambda)$ and $\bar{z}(\lambda)$ are the color matching functions,

$$X = k \int_{380nm}^{780nm} S(\lambda)R(\lambda)\bar{x}(\lambda)d\lambda \tag{5}$$

$$Y = k \int_{380nm}^{780nm} S(\lambda)R(\lambda)\bar{y}(\lambda)d\lambda \tag{6}$$

$$Z = k \int_{380nm}^{780nm} S(\lambda)R(\lambda)\bar{z}(\lambda)d\lambda \tag{7}$$

where

$$k = 100 / \int_{380nm}^{780nm} S(\lambda)\bar{y}(\lambda)d\lambda \tag{8}$$

After the chromatic adaptation transform, the RGB values corresponding to the new XYZ values are calculated by the inverse procedures of Eqs. 2 to 4, then set as standard matrix $[A]$. The color relationship between the spectrophotometer and the camera is found by using the third-order polynomial regression for the red, green and blue components separately, and the

regression matrix $[C]$ is determined as follows:

$$[C]=[A]pinv[F] \tag{9}$$

where

$$[F]=\left[1,R,G,B,RG,GB,BR,R^2,G^2,B^2,RGB,R^3,G^3,B^3,RG^2,RB^2,GR^2,GB^2,BR^2,BG^2\right]^T \tag{10}$$

and "R", "G", and "B" are the respective RGB values of the color checkers captured by the camera. The corrected RGB values are obtained from Eq. 11. Here [K] presents the RGB values captured from any image that expanded into a format such as the original matrix [*F*]. The calculation processes of the CMCCAT2000-corrected RGB values of the digital camera are obtained from reference 15.The corrected RGB values of the color checkers are then transferred into CIE XYZ values and arrayed as a matrix, $[\beta]$.

$$[\text{Corrected RGB}]=[\text{C}][\text{K}] \tag{11}$$

Finally, a transform matrix $[M]$ between the spectrophotometer and the camera is obtained as follows:

$$[M]=[\alpha]pinv[\beta] \tag{12}$$

For each pixel in any image captured by the camera, the RGB values multiplied by the regression matrix $[C]$ and the corresponding XYZ values are calculated using Eqs. 2 to 4. The estimated spectra in the visible light region (380nm to 780nm) are obtained by

$$[Spectra]_{380-780nm}=[E][M]\begin{bmatrix}X\\Y\\Z\end{bmatrix} \tag{13}$$

Esophageal cancer presents a spectrum of different diatheses. A precise assessment for individualized treatment depends on the accuracy of the initial diagnosis. Detection relies on comprehensive and accurate white-light cystoscopy. In addition to its invasive nature and the potential risks related to the method, white-light cystoscopy has limitations, including difficulties in flat lesion detection, precise tumor delineation to enable complete resection, inflammation and malignancy differentiation, and grade and stage determination. The resolution of these problems depends on the surgeon's ability and experience with available technology for visualization and resection. In this study, we used multi-spectral imaging technology combined with phase contrast microscopy to analyze ECCs at various stages using a single-cell array chip. We found from the spectral characteristics of single cell that the cell spectra at the different cancer stages demonstrate a change in the cell's composition.

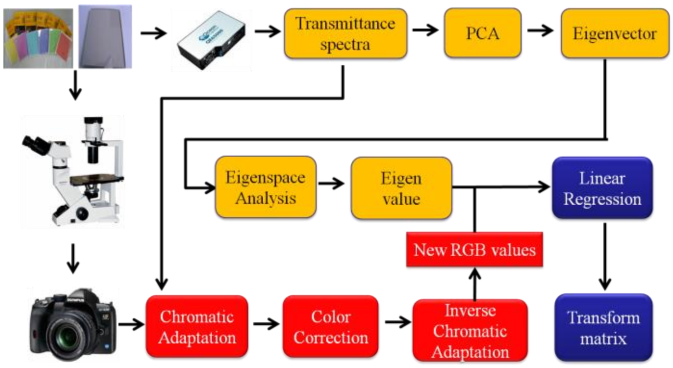

Figure S2 Schematic diagram of the proposed method used in estimating the spectral transmittance of each pixel of an image using a DSLR camera.

S2. Selection of invasion cells by transwell invasion chamber

Subpopulations from the CE81T ECC line were selected using the membrane invasion culture system (MICS) or BD BioCoat™ Matrigel™ Invasion Chamber (MA, USA). Briefly, cells were suspended in DMEM containing 10% FBS and seeded into the wells. After incubation at 37 °C for 72 h, the inserts were removed. The cells that had invaded the membranes and had attached to the lower-chamber compartments were harvested and allowed to proliferate for a second round of selection. For MICS selection, the sublines of the first-round selection in the upper and lower well chambers were designated as CE81T1-0 and CE81T1-1, respectively; the sublines from the second, third, and fourth rounds of selection were designated as CE81T1-2, CE81T1-3, and CE81T1-4, respectively. The parental line in the first series was designated CE81T1. For BD BioCoat™ Matrigel™ Invasion Chamber selection, the sublines of the first-round selection in the upper and lower well chambers were designated CE81T2-0 and CE81T2-1, respectively, and the sublines from the second, third, and fourth rounds of selection were designated CE81T2-2, CE81T2-3,and CE81T2-4, respectively. The parental line in the second series was designated CE81T2.

MICS was used to measure the invasion capacity of each cell line. Assays were performed using polycarbonate membranes (Falcon HTS Fluoro Blok™ insert, BDBiosciences, Franklin Lakes, NJ, USA). Invasion assay was performed using membranes with uniformly-coated reconstituted basement gel (Matrigel, BD Biosciences, Bedford, MA, USA). The insert was placed in a 24-well culture dish (Falcon) containing DMEM and 10% NuSerum (BD Biosciences). In each well, $5{\times}10^4$ cells were resuspended in DMEM containing 10% NuSerum, and then seeded into the upper wells of the chamber. After 36 h of incubation at 37 °C, cells that had migrated or had invaded the membrane were stained with the fluorogenic compound 4,6-diamidino-2-phenylindole (DAPI, Sigma, St. Louis, MO, USA). Invading cells were counted manually using three random microscopic fields per well. The DAPI fluorescence of nuclei was visualized by excitation at 330–385 nm with a 420 nm barrier filter. Images were captured using a Nikon inverted fluorescence microscope (Nikon ECLIPSE TE300, Tokyo,

Japan) with attached camera at ×100 magnification and processed using ImagePro PlusVersion 5.0 software (Media Cybernetics, MD, USA). Experiments were repeated in triplicate.
